# Effective intercalation of zein into Na-montmorillonite: role of the protein components and use of the developed biointerfaces

**DOI:** 10.3762/bjnano.7.170

**Published:** 2016-11-18

**Authors:** Ana C S Alcântara, Margarita Darder, Pilar Aranda, Eduardo Ruiz-Hitzky

**Affiliations:** 1 Instituto de Ciencia de Materiales de Madrid, CSIC, Cantoblanco, 28049-Madrid, Spain; 2 present address: Universidade Federal do Maranhão, Departamento de Química – PPGQuim, LIM-Bionanos, 65080-805, São Luís, MA, Brazil

**Keywords:** biohybrids, biointerfaces, bionanocomposites, montmorillonite, zein

## Abstract

Biohybrid materials based on the intercalation of zein, the major storage protein in corn, into sodium-exchanged montmorillonite were prepared following two synthesis strategies. The first one made use of zein dissolved in 80% (v/v) ethanol/water solution, the usual solvent for this protein, while the second method is new and uses a sequential process that implies the previous separation of zein components in absolute ethanol. This treatment of zein with ethanol renders a soluble yellow phase and an agglomerate of insoluble components, which are able to intercalate the layered silicate when an aqueous dispersion of montmorillonite is added to the ethanol medium containing both phases. The diverse steps in this second route were investigated individually in order to understand the underlying mechanism that drives to the intercalation of this complex hydrophobic biomacromolecule into the hydrophilic interlayer space of sodium-exchanged montmorillonite. In addition to physicochemical characterization of the resulting materials, these biohybrid interfaces were also evaluated as biofillers in the preparation of diverse ecofriendly nanocomposites.

## Introduction

Organic–inorganic hybrids are composed of organic and inorganic units that interact at the molecular scale, and the characteristics of these subunits determine a broad range of properties of the hybrid relevant for many applications [1]. Particularly, biological species can be employed in the preparation of these materials, giving rise to biohybrids, which represent a growing field of research addressed to produce advanced functional materials [2]. Many studies have demonstrated that even large molecules, such as polypeptides and proteins, intercalate into montmorillonite and other smectite clay minerals, producing biohybrid materials [2]. Montmorillonite is a 2:1 phyllosilicate characterized by a colloidal particle size, high specific surface area and large cation exchange capacity (CEC) around 70–100 milliequivalents/100 g of clay. Structurally each silicate layer is formed by the repetition of a central octahedral alumina sheet sandwiched by two tetrahedral silica sheets [3]. Isomorphic substitutions in the octahedral and, partially, in the tetrahedral sheets result in a net negative charge of the layers that is compensated by cations (typically Na^+^ and Ca^2+^) located in the interlayer region [3]. These interlayer cations are exchangeable by treatment with diverse cationic species, being the reason of its extensive use in the development of hybrid materials by ion-exchange intercalation reactions. Since 1950 when Talibudeen reported on the intercalation of gelatin into montmorillonite [4], other biohybrids also based on the assembly of smectite clays and proteins (e.g., bovine serum albumin, gelatin, casein or soy) have been vastly studied [5–10]. However, protein adsorption on montmorillonite clay can be considered a complex process in which the structural stability of the protein, the ionic strength, the pH value as well as the surface properties can influence the affinity of the biomolecule toward the inorganic interface. In addition to the particular characteristics of each protein, the structural size and proportion of hydrophobic residues may be also a key factor in order to achieve their intercalation in montmorillonite [11–12]. Thus, depending on the type of protein involved, it is possible to obtain different interaction mechanisms between the clay and the biomacromolecule, generating the need to investigate possible interactions that can occur in less-studied proteins.

Zein is the major storage protein of corn and an important source of protein in the human diet either through direct consumption or through consumption of animals whose feed is based on corn, such as poultry or swines [13]. Although zein is known since 1821 [14–15], there is only recent interest in this protein focusing on its potential technological use [16–17]. Zein is insoluble in water but soluble in aqueous ethanol (60–95% (v/v)), aqueous solution at pH > 11, some organic polar solvents (e.g., propylene glycol and acetic acid) and certain anionic detergents [16,18–19]. The solubility is attributed to the non-polar amino acid residues of the protein, such as valine, leucine, proline, isoleucine, alanine, and phenylalanine, which confer a hydrophobic character to the protein [16]. Therefore, a good knowledge of structural and solubility properties of zein becomes essential for the preparation of materials based on this protein. In this sense, there are reports on zein–montmorillonite composite materials prepared by thermo-plasticization and blown-extrusion techniques [20–21], or from protein solved in ethanol/water mixtures [22]. However, in these examples, organoclays containing alkylammonium surfactant species [20,22] or polyethylene glycol as plasticizer [21] were required to produce the zein-based materials. Nevertheless, the process of formation of zein–montmorillonite biohybrids making use of sodium-exchanged montmorillonite (Na-montmorillonite) and the possible mechanism that leads to them have not been described so far. With regard to this, the complex structure of zein and the role of the amino acids in its composition have to be considered, as well as the specific conformation of this protein, in order to understand a mechanism that may drive to the biohybrid formation.

In this paper, a systematic study on the preparation of zein–montmorillonite biohybrids is reported, focusing on the control of solubilized zein for an effective intercalation of the protein into Na-montmorillonite. In order to investigate the underlying mechanism of zein intercalation, the structure and features of the synthesized biohybrids were also analyzed. Zein-based biohybrids were further tested as reinforcing fillers of other biopolymer matrixes to probe their usefulness in the development of “fully” ecofriendly bioplastics. In fact, organoclays prepared by intercalation of biomolecules such as lipids or proteins have been recently reported [23–26], resulting in so-called bio-organoclays useful as fillers in the preparation of bionanocomposites or as biointerfaces for adsorption of biological species. In the present case, the incorporation of the biofillers intends to improve their compatibility with the polymer matrix, while keeping the biocompatible character of the material, and additionally incorporating interesting properties, such as barrier properties, as reported for other bio-organoclays used in reinforced bioplastics [25,27–28].

## Results and Discussion

### Characterization of zein in (80% v/v) ethanol solution and absolute ethanol

**Figure 1:**
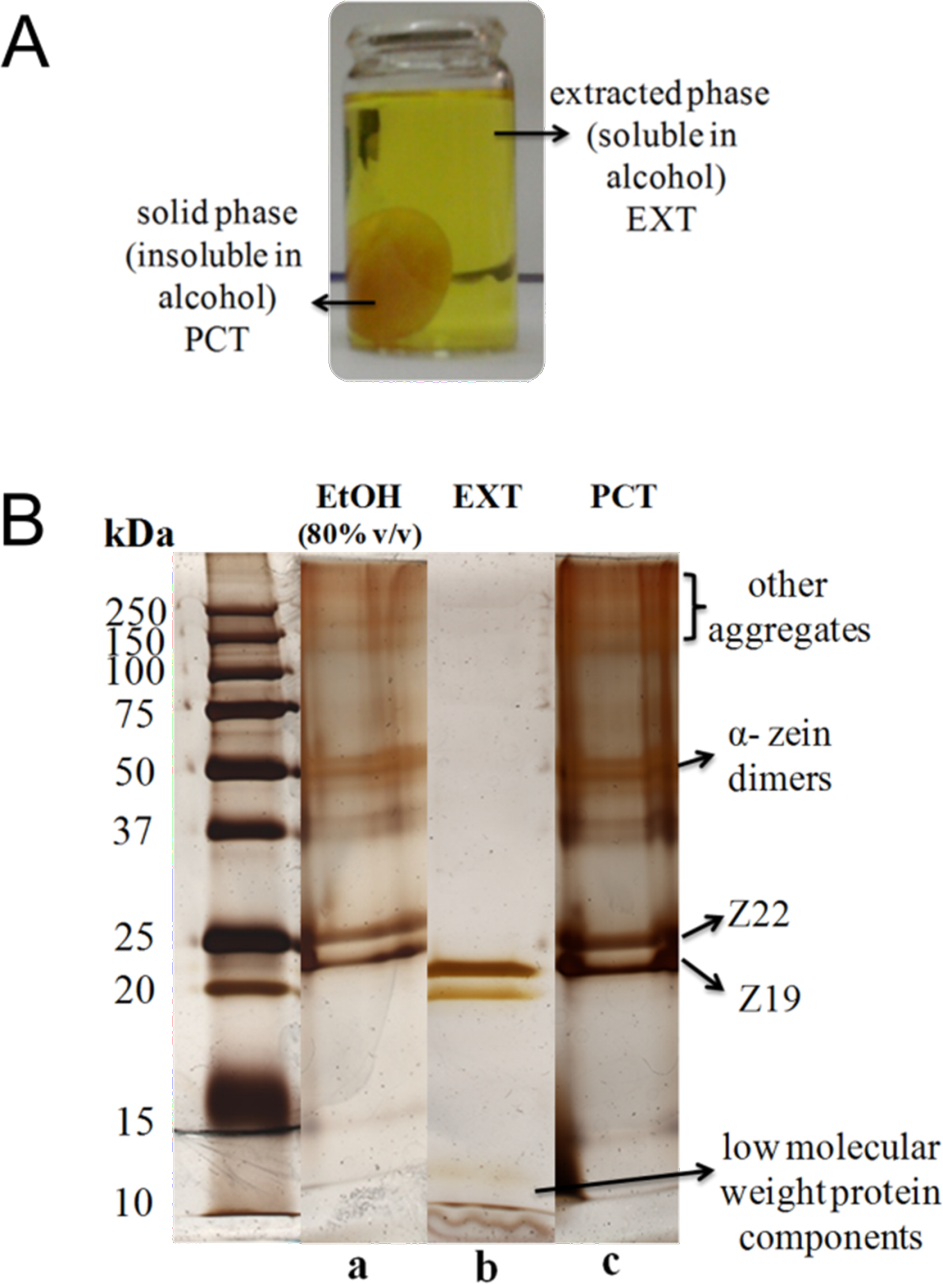
(A) Picture showing the separation phenomenon observed when zein protein is dispersed in absolute ethanol, and (B) SDS-PAGE of zein in 80% (v/v) ethanol/water solution (a) and of the EXT (b) and PCT (c) zein fractions separated in absolute ethanol. PCT was dissolved in 80% (v/v) ethanol/water for the analysis. The electrophoretic gel was silver stained.

Zein is not soluble in water or pure alcohol. Ethanol/water mixtures of 80% (v/v) are the most commonly used solvent. However, it was observed in this work that a separation process of different components of zein in a soluble phase (EXT) and a precipitate (PCT) occurs in pure ethanol (Figure 1A), as detailed in the Experimental section. These phases show different textural characteristics as observed by FE-SEM (Supporting Information File 1, Figure S1). Colorimetric tests using a ninhydrin spray solution as revealing agent confirmed the presence of protein in the extracted liquid phase, showing a purplish color resulting from reaction between ninhydrin and free amino groups from amino acids of solubilized zein (Supporting Information File 1, Figure S2).

The molecular weight of the protein was investigated by electrophoresis (SDS-PAGE) as presented in Figure 1B. Zein solubilized in 80% v/v ethanol solution (Figure 1B, lane a) gives rise to two bands at approximately 23 and 25 kDa indicative of the α-zein conformation, which correspond to Z19 and Z22 proteins, respectively [29]. This SDS-PAGE gel also shows bands around 50 kDa that reveal the presence of α-zein dimers [30–31], as well as bands at higher molecular mass (around 150 and 250 kDa) corresponding to other protein aggregates, such as trimers, tetramers and/or oligomers.

Electrophoresis measurements conducted in the PCT and EXT phases revealed that the protein pattern of PCT (Figure 1B, lane c) is very similar to that of neat zein (Figure 1B, lane a), with bands of α-zein and its dimers at approximately 21–25 kDa and 50 kDa, respectively. An intense band set of protein aggregates is also observed between 150 kDa and 250 kDa. The SDS-PAGE of the EXT phase presents a band at around 10 kDa together with those corresponding to α-zein (Figure 1B, lane b). This 10 kDa band, also reported by other authors [31], could be related to ethanol-soluble protein components associated with the xanthophyll pigments responsible for the yellow color of zein, such as lutein and zeaxanthin, which are located in the core of the triple-helical segments strongly linked to the Z19 monomer [32].

The FTIR spectra in the 4000–500 cm^−1^ region of zein, EXT and PCT (Supporting Information File 1, Figure S3A) show the bands of amide A (3600–3100 cm^−1^), amide I (1665–1655 cm^−1^), and amide II (1540–1530 cm^−1^) of the protein. The spectrum of PCT (Supporting Information File 1, Figure S3A, spectrum c) is very similar to that of EXT (Supporting Information File 1, Figure S3A, spectrum b), except that the latter shows more evidently the presence of a shoulder at 1742 cm^−1^ attributed to the ν_C=O_ vibrations of carboxylic groups present in the protein structure [33]. CP-MAS ^13^C NMR (Supporting Information File 1, Figure S3B) spectra of zein and the two fractions separated from ethanol are complex and very similar to each other, presenting signals between 173–175 ppm due to carbonyls present in the peptide groups in both the main chain and the protein side chains. The signals at 128 ppm, those from 45 to 70 and those from 15 to 45 ppm are assigned to amino acid aromatic side chains, α-carbons linked to amino groups, and carbons from the amino acid aliphatic side chains, respectively [33].

Thus, these studies indicate that although zein cannot be dissolved in pure ethanol, a separation process of different components of the protein occurs in this medium corresponding to the soluble (EXT) and insoluble zein fractions (PCT), where EXT phase is composed mainly by monomers and other protein components of low molecular weight, while PCT phase is formed by protein fractions of higher molecular mass.

### Zein–montmorillonite biohybrids

A protocol that uses zein in its usual solvent, 80% (v/v) ethanol/water, and the clay dispersed in the same medium was firstly tried for the preparation of zein–montmorillonite biohybrids (synthesis 1). In an alternative synthetic approach (synthesis 2) the clay was dispersed in water, favoring the formation of a swollen phase, and zein was treated with pure ethanol to provoke its segregation in two phases. Then both systems were mixed until reaching a content of 80% (v/v) ethanol/water and left to evolve to equilibrium. The amount of zein adsorbed on montmorillonite (MMT) through these two synthetic procedures starting from systems with variable amounts of zein in contact with a defined amount of clay was deduced by CHNS chemical analysis and the results are summarized in Table 1. The values of zein adsorption on MMT prepared by synthesis 2 are almost two times higher than those resulting from synthesis 1, which suggests a different adsorption mechanism in each procedure. In both cases the adsorption increases rapidly at low protein concentration, and then reaches a constant value of approximately 11.0 g and 40.0 g of adsorbed zein per 100 g of MMT in synthesis 1 and 2, respectively. This plateau region at zein equilibrium concentration above 0.25 and 2.0 g·L^−1^ in each case can be better observed from the two adsorption curves in Figure S4 (Supporting Information File 1). At higher equilibrium concentration, the amount of adsorbed zein increases rapidly, probably because in highly concentrated solutions zein is present as aggregates that adsorb on the silicate, as observed in biohybrids of zein and fibrous clays [25].

**Table 1:** Biohybrids of the Z-MMT_S1 and Z-MMT_S2 series, prepared by adsorption of zein from 80% (v/v) ethanol/water solution and from zein segregated phases in absolute ethanol, respectively. The protein content was determined by CHNS chemical analysis.

mass of zein (in g) in contact with clay (100 g clay)	Z-MMT_S1 biohybrids codes	adsorbed zein (g zein/100 g MMT)	Z-MMT_S2 biohybrids codes	adsorbed zein (g zein/100 g MMT)

10.0	Z-MMT_S1-7	7.74	Z-MMT_S2-9	9.25
20.0	Z-MMT_S1-9	9.13	Z-MMT_S2-14	14.0
40.0	Z-MMT_S1-9.5	9.52	Z-MMT_S2-20	20.3
66.6	Z-MMT_S1-10	10.3	Z-MMT_S2-26	26.2
100.0	Z-MMT_S1-11	10.8	Z-MMT_S2-35	35.5
166.0	Z-MMT_S1-14	14.6	Z-MMT_S2-37	37.5
333.3	Z-MMT_S1-20	20.8	Z-MMT_S2-40	39.5
500.0	Z-MMT_S1-27	27.4	Z-MMT_S2-46	46.6

**Figure 2:**
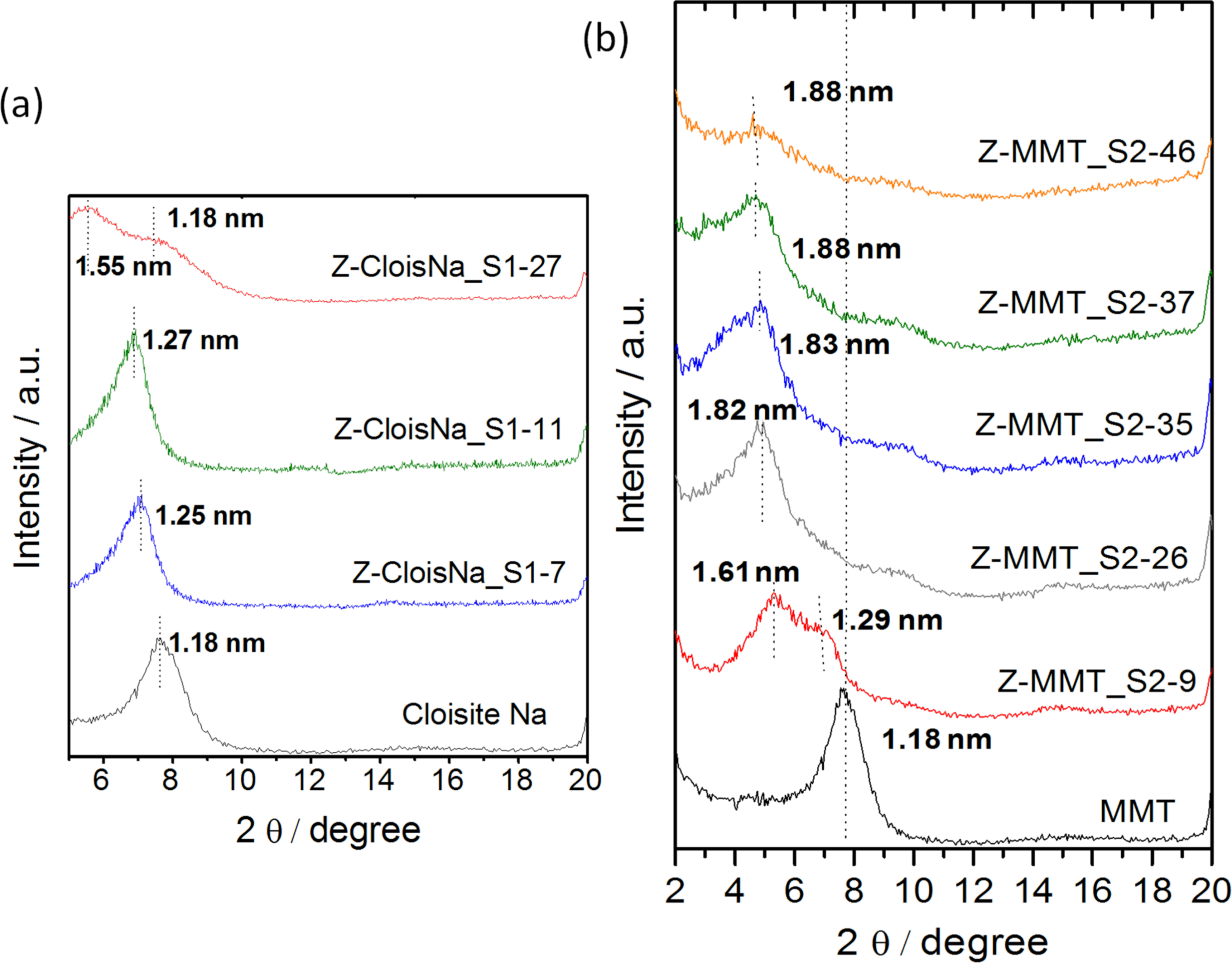
XRD patterns of (a) Z-MMT_S1 and (b) Z-MMT_S2 biohybrids.

The XRD patterns of the Z-MMT_S1 biohybrid materials with low adsorbed protein content show the (001) reflection peak at a 2θ value close to that of pristine MMT (Figure 2a). The diffractogram of Z-MMT_S1-27, with the highest zein content, shows two reflections at low 2θ angles, one corresponding to a *d*_00l_ value of 1.18 nm, similar to that of MMT, and a second broad peak centered at 1.55 nm that can be related to the presence of an intercalated phase. Taking into account the thickness of the silicate layer of 0.96 nm [34], the interlayer distance (∆_dL_) gives a value of 0.59 nm, which is lower than the dimensions of the zein monomer, considering that the α-helix monomer of zein has a thickness of approximately 1.2 nm [35]. A possible explanation could be related to a partial intercalation of zein, affecting only the edges of the clay particles via ion-exchange reaction of Na^+^ by protonated glutamine groups in the loops of α-helix zein molecules. Anyway, the presence of two (001) reflections in the diffractogram suggests the presence of mixed phases in the materials prepared under these synthesis conditions. The resulting biohybrids are probably formed with most of the protein molecules situated just at the external surface of the sodium montmorillonite, concluding that zein cannot be intercalated effectively in MMT by this method, as already reported by Park and co-authors in studies on zein–montmorillonite biohybrids processed by electrospinning [36].

On the other hand, the XRD patterns of Z-MMT_S2 biohybrids (Figure 2b) confirm the intercalation of zein in the clay interlayer space by the shift of the 001 peak towards lower 2θ values. Although the XRD pattern of Z-MMT_S2-9 shows a broad peak, it is possible to evidence two peaks corresponding to basal spacings of 1.61 and 1.29 nm, which suggest mixed phases with and without intercalated protein, respectively. The value of *d*_00l_ increases with zein content and can reach a value of around 1.88 nm for zein contents higher than 25 g of protein per 100 g of MMT. The increase of the interlayer distance can be estimated to about 0.92 nm for Z-MMT_S2-37 and Z-MMT_S2-46 biohybrids, this latter having the highest content in zein. In this case, the intercalated protein is probably distorting its structure to become accommodated in the interlayer region. Similar results were reported for other proteins intercalated in sodium montmorillonite, such as bovine serum albumin (BSA) [5]. Therefore, the new route 2 seems more effective to achieve the incorporation of zein molecules into the intracrystalline space of sodium montmorillonite.

**Figure 3:**
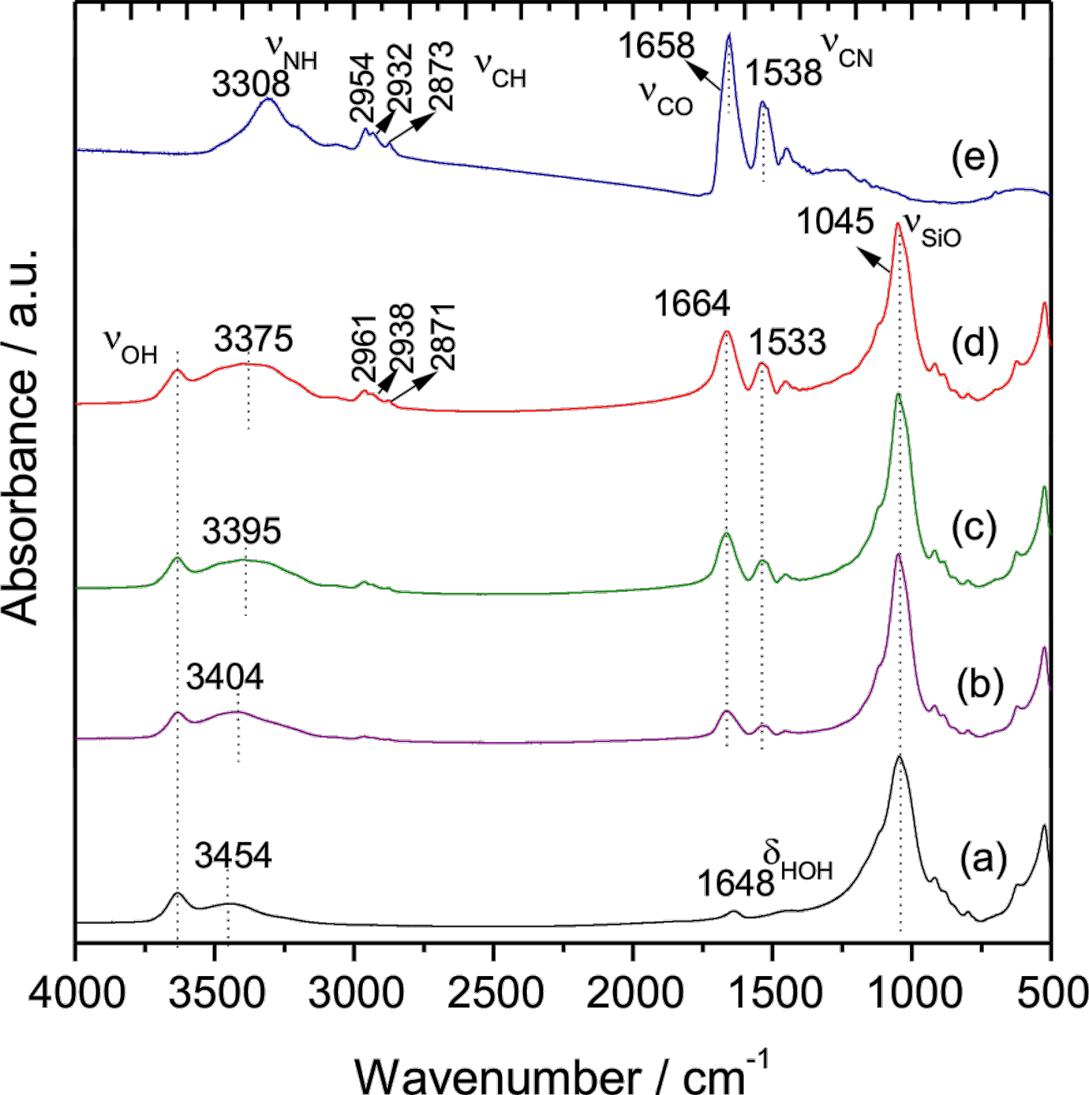
FTIR spectra in the 4000–500 cm^−1^ region of (a) starting MMT, (b) Z-MMT_S2-9, (c) Z-MMT_S2-26 and (d) Z-MMT_S2-46 biohybrid materials, and (e) pristine zein.

The infrared spectra of pristine MMT, zein, and Z-MMT_S2 biohybrids which contain 9.25, 26.2 and 46.6 g of protein per 100 g of MMT, respectively, are shown in Figure 3. The IR bands at 3634, 1648 and 1045 cm^−1^ are assigned to ν_OH_ modes of Al,Mg(OH) and δ_HOH_ modes of water molecules in the clay and characteristic ν_Si–O–Si_ vibration modes of the aluminosilicate, respectively (Figure 3a). Other bands that can be attributed to the intercalated protein are also observed in the spectra of the biohybrids. The frequency of the band corresponding to the ν_CO_ vibration mode of amide I that appears at 1658 cm^−1^ in pristine zein (Figure 3e) is shifted toward higher frequency values, reaching wavenumber values of 1664 cm^−1^ in the biohybrids (Figure 3b–d). This shift may be a consequence of the perturbation introduced in the amide group by interactions between the involved protonated amino groups and the negatively charged sites in the clay structure. Similar results involving changes in the amide-I band of zein were reported by Ozcalik and Tihminlioglu [37] in bionanocomposites based on organomodified montmorillonite. The band ascribed to the ν_NH_ vibration mode of the amide-A groups in zein also appears at higher wavenumber in the biohybrids, where the frequency depends on the amount of intercalated protein. This observation points out to the existence of hydrogen bonding interactions between such groups of zein and the interlayer water molecules in the clay [38]. The presence of interactions between the protein and the MMT clay is also confirmed by the displacement to lower wavenumbers of the ν_CN_ amide-II band of zein at 1538 cm^−1^, appearing in the biohybrids at 1533 cm^−1^.

**Figure 4:**
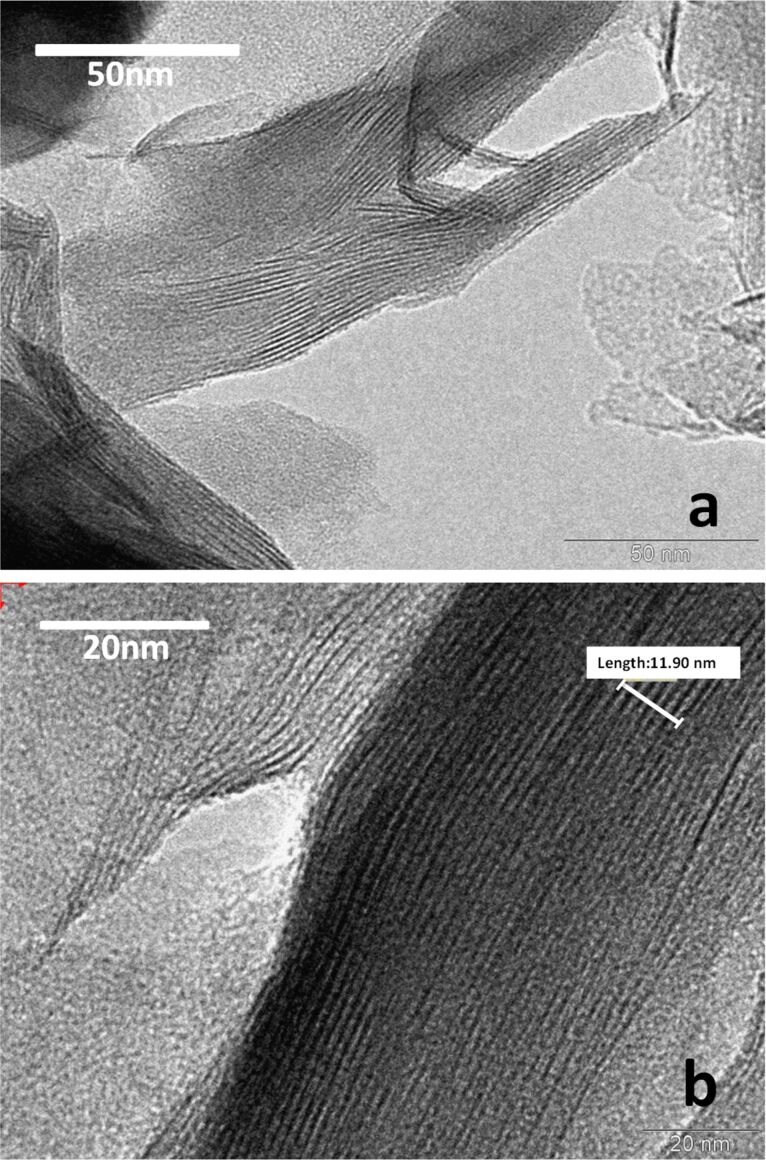
TEM images of the Z-MMT_S2-46 biohybrid sample. In b) the region used to estimate the basal space distance is signaled (seven sheets).

The Z-MMT_S2-46 intercalation compound, with the highest content in zein, was chosen for characterization by TEM microscopy (Figure 4). These images show the presence of the characteristic platelets of montmorillonite tactoids, which confirm that the intercalation of zein does not affect the intrinsic organization of the layered clay. By a calculation using an average of seven sheets (measurement performed by the microscope software, Figure 4b), it was found a basal spacing average of 1.7 nm in this TEM image, close to that deduced from the XRD patterns that clearly confirms the intercalation of the protein.

### Intercalation mechanism of zein in MMT from protein segregated phases in absolute ethanol

In order to understand the mechanism underlying the intercalation process in synthesis 2, the interaction of MMT with each zein phase segregated in absolute ethanol was investigated individually (Figure S5a, Supporting Information File 1). Thus, the extracted phase (EXT), after removal of the precipitate fraction (PCT), was used to prepare biohybrids by addition of an aqueous clay suspension, resulting in a series of materials denoted as EXT–MMT biohybrids (Supporting Information File 1, Figure S5a, route 1). On the other hand, biohybrids based on PCT and MMT were prepared by directly mixing the MMT aqueous suspension and PCT re-suspended in pure ethanol, obtaining the so-called PCT–MMT biohybrids (Supporting Information File 1, Figure S5a, route 2). In both cases, the biohybrids were formed in a final liquid phase of 80:20 (v/v) ethanol/water, i.e., similar to that used in synthesis 2.

These EXT and PCT phases were obtained from three different initial amounts of zein in pure ethanol: 20, 100 and 500 g of protein per 100 g of MMT. The amount of protein adsorbed on MMT in each case, determined by CHNS chemical analysis, was 3.32, 10.2, and 15.8 g per 100 g of MMT in the EXT–MMT biohybrids, and 18.0, 43.2, and 65.5 g per 100 g of MMT in the PCT–MMT materials. These results reveal that the biohybrids based on EXT show a lower amount of adsorbed protein than those prepared from PCT. This fact may be related to the adsorption of zein oligomers present in the PCT fraction, as shown by SDS-PAGE (Figure 1B). Adsorption of the components from EXT on MMT is confirmed from analysis by UV–vis spectroscopy of the supernatant separated after formation of the EXT–MMT biohybrids (Supporting Information File 1, Figure S6), which shows clearly the decrease of intensity in the bands ascribed to protein and carotenoid components with respect to those in the EXT phase.

**Figure 5:**
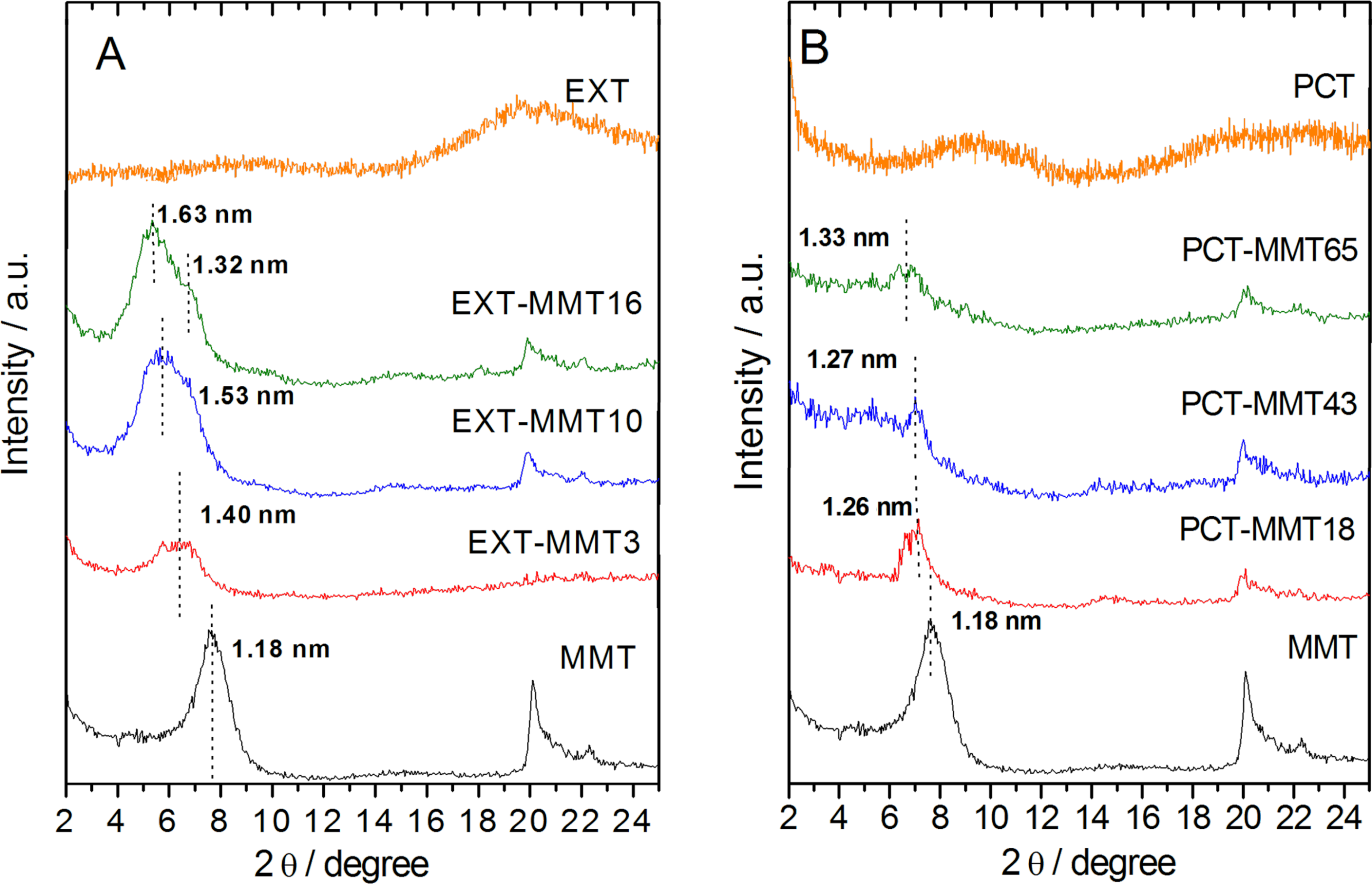
XRD patterns of the biohybrids prepared from (A) the extracted (EXT) and (B) the precipitate (PCT) fractions separated from different amounts of zein in pure ethanol.

The X-ray diffractograms of the EXT–MMT samples (Figure 5a) show a progressive increase of the interlayer distances as the amount of adsorbed protein increases, reaching a basal spacing value of 1.63 nm for the EXT-MMT16 sample. On the other hand, the d_00l_ values determined in the PCT–MMT biohybrids range between 1.26 and 1.33 nm (Figure 5b), indicating that only a negligible intercalation took place in this case. The protein adsorbed from the PCT phase is rather located at the external surface of the clay.

The key to understanding such differences in the adsorption behavior from these two zein phases is the nature of each protein fraction. Natural zein shows two reflections at approximately 2θ = 9.3° and 20.4° in its XRD pattern, attributed to interhelix packing structure and zein α-helix backbone, respectively [21]. The EXT phase shows only a reflection around 2θ = 20.4° (Figure 5a), indicating the absence of interhelix packing, probably because the arrangement in molecular aggregates is not favored in pure ethanol, as shown by SDS-PAGE (Figure 1B, lane b). This would favor the intercalation of this phase in MMT. Conversely, in addition to a broad signal at 2θ = 20–25° that reveals the presence of zein α-helix structure, the diffractogram of the PCT phase (Figure 5b) shows a peak at 2θ = 9.3° ascribed to interhelix-packing domains [21], as also corroborated by SDS-PAGE (Figure 1B, lane c). The presence of these molecular aggregates in the PCT fraction, due to interhelix packing, could hinder the incorporation of zein molecules between the MMT layers, as shown in Figure 5b.

**Figure 6:**
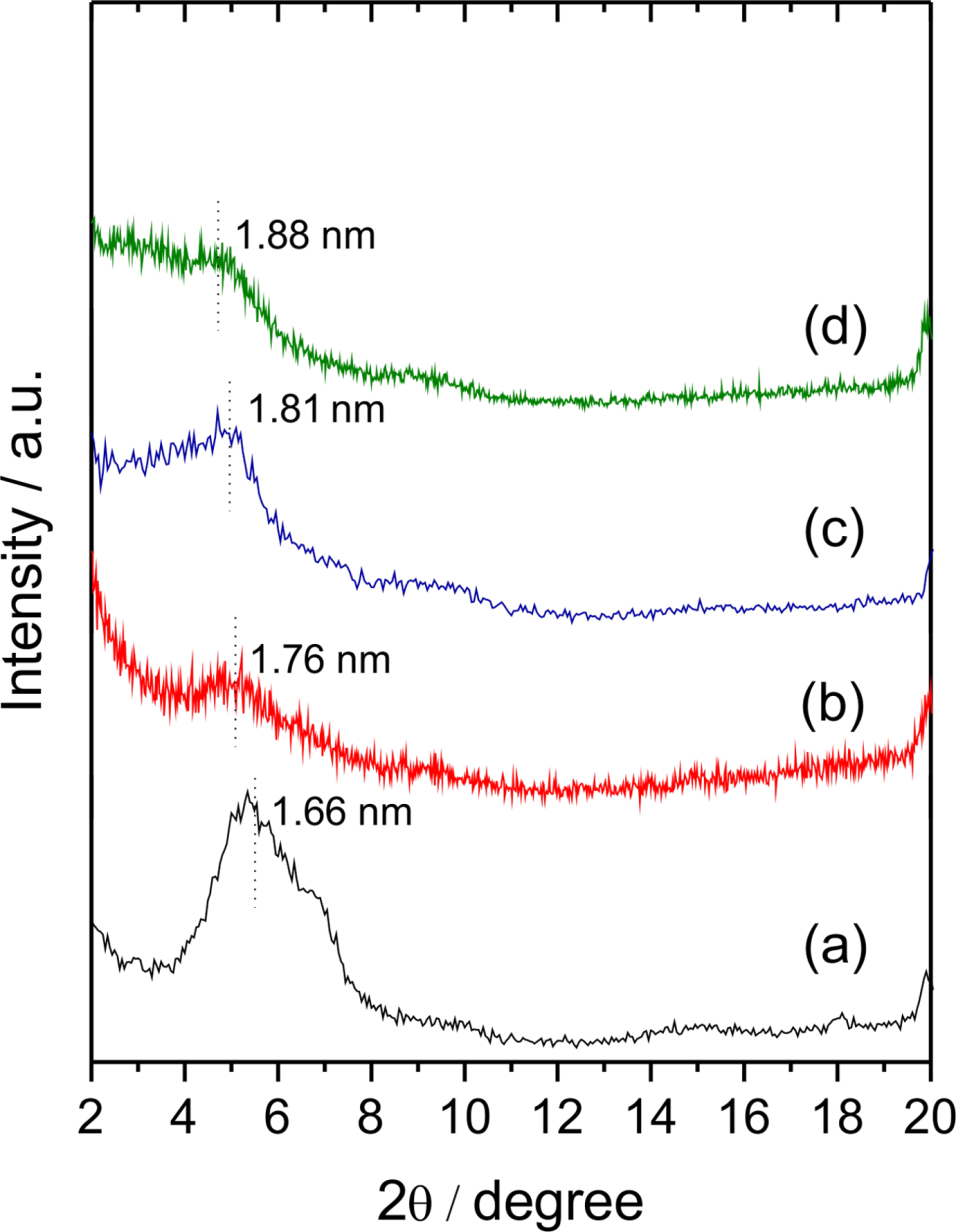
XRD patterns of (a) EXT-MMT16 biohybrid and the EXT–MMT/PCT biohybrids prepared with different PCT phases recovered from (b) 0.06, (c) 0.3 and (d) 1.5 g of zein in 80 mL of pure ethanol.

Considering that the EXT phase seems to play a significant role in the mechanism of zein intercalation between the layers of montmorillonite, EXT–MMT biohybrids (in aqueous dispersion) were employed as substrate for the incorporation of the PCT phase (in pure ethanol) giving rise to a series of biohybrids named as EXT–MMT/PCT (Supporting Information File 1, Figure S5b). The XRD patterns of these biohybrids are displayed in Figure 6. As observed, the characteristic 001 rational order peak in the starting EXT-MMT16 material containing 15.8 g of zein per 100 g of MMT (Figure 6a) is shifted towards lower 2θ values in the EXT–MMT/PCT biohybrids (Figure 6b–d). These basal spacing values slightly increase as the PCT content increases in the dispersion, reaching a maximum value of 1.88 nm (Figure 6d). This basal spacing value is similar to that of the Z-MMT_S2-46 biohybrid (1.88 nm), which is the biohybrid prepared by synthesis 2 route using the highest amount of zein (Figure 2b). These results are in agreement with those reported by Weiss [11], which revealed that intercalation of several proteins, such as salmin, serum and egg albumin, generally never surpasses a basal spacing of about 1.8 nm, independently of the initial protein concentration. However, in this case, although the intercalation of PCT moiety in the EXT-MMT16 hybrid can be confirmed by XRD studies, it cannot be ruled out that some PCT fractions could be also adsorbed at the external surface of the mineral. Thus, it can be inferred from this study that the mechanism of zein intercalation in MMT from the zein phases separated in ethanol could be associated with two main stages: i) the formation of a bio-organoclay based on MMT and ethanol-soluble components of zein, i.e., monomers and other protein components of low molecular weight, which can be more easily incorporated into the water-swollen MMT; ii) the intercalation of PCT constituents into the clay interlayer space possibly ascribed to the cooperative role of the already adsorbed components of zein in the biohybrid. This proposed mechanism could explain the ability of the hydrophobic protein to penetrate into the interlayer region of sodium montmorillonite following the “synthesis 2” approach proposed in this work.

### Zein-layered clays as nanofillers in biopolymer films

**Figure 7:**
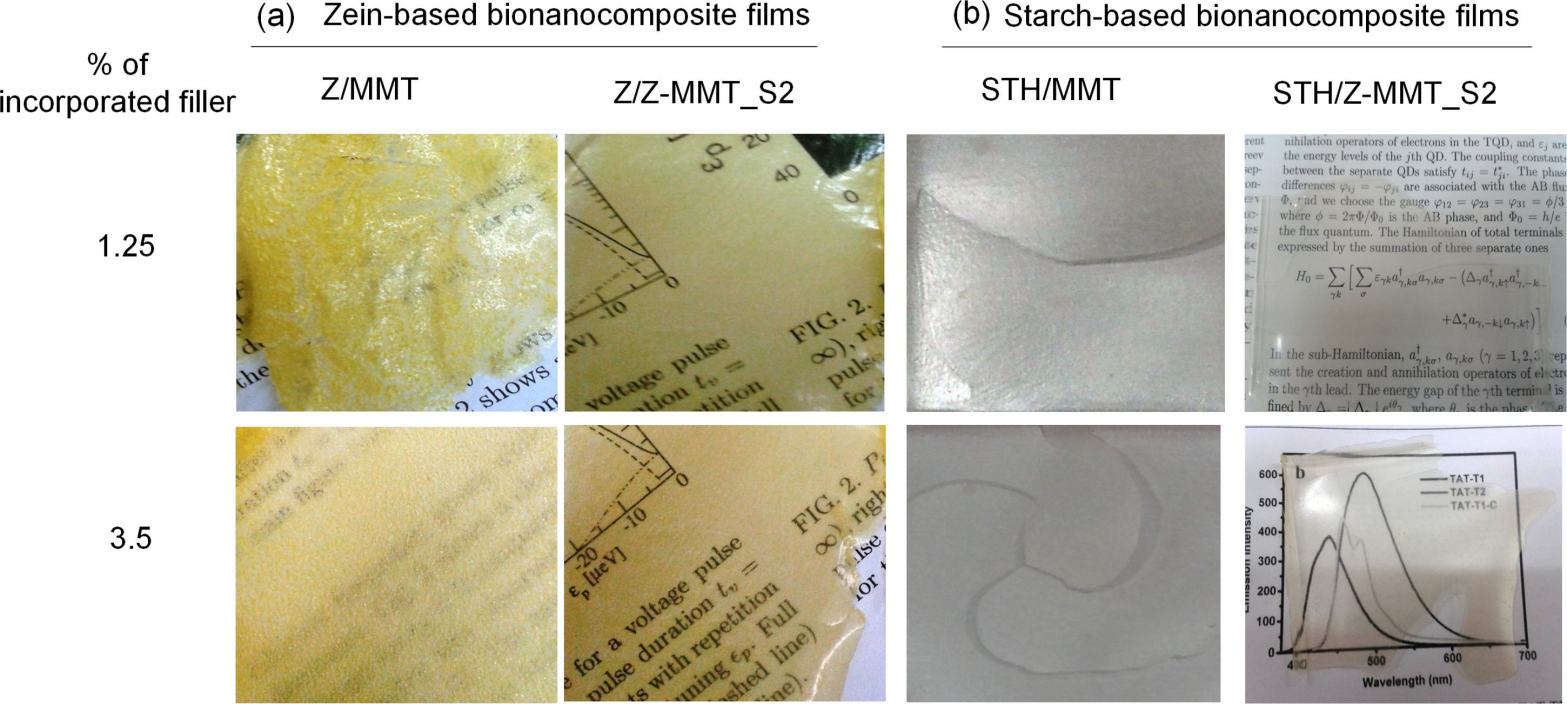
Photographs of (a) zein and (b) starch bionanocomposite films loaded with pure MMT or Z-MMT_S2-46 biohybrid.

The biocompatible character of the developed bio-organoclays makes them an environmentally friendly alternative to alkylammonium-based organoclays for the use as nanofiller of bioplastics. Thus, the intercalation compound Z-MMT_S2-46 was tested as biofiller of zein (Z) and starch (STH) matrices and compared to neat MMT. Bionanocomposite films with 1.25 and 3.50% of biofiller and pure biopolymer films were prepared by casting methods (Figure 7). For this it is necessary to add a certain amount of glycerol as plasticizer component in the blank films to reduce their high brittleness. The light transmittance of zein bionanocomposites was higher for films containing Z-MMT_S2, but in the starch materials its effect was similar to that of MMT (Supporting Information File 1, Figure S7). Self-standing MMT-modified zein films show great opacity and an inhomogeneous aspect ratio (Figure 7a), due to a poor compatibility between the hydrophilic clay and the hydrophobic zein matrix. In contrast, Z/Z-MMT_S2 bionanocomposite films present a higher homogeneity and transparency (Figure 7a), confirming a good dispersion of the biohybrid within the zein matrix. Interestingly, all the bionanocomposite films based on starch appear very homogenous (Figure 7b), but those loaded with neat MMT are very brittle and exhibit fractures. This behavior is likely due to the absence of plasticizer, commonly used to increase the flexibility of the resulting material. Conversely, starch films loaded with 1.25% (w/w) of Z-MMT_S2 biohybrid do not exhibit fractures even without the addition of plasticizer, indicating the good compatibility of the biohybrid and the polysaccharide matrix.

Tensile-stress studies were carried out for the zein and starch bionanocomposite films. Those films loaded with neat MMT and 3.5% (w/w) Z-MMT_S2 biohybrid were excluded from this study due to their high brittleness. Similar behavior was observed for pure zein and starch films, and in these cases glycerol was added as plasticizer in order to use them as reference.

The Young´s modulus of the Z/Z-MMT_S2 film is 1.2 GPa, about 2.5 times higher than that of the unmodified film of zein (0.5 GPa), being analogous to that reported by Nedi et al. [21] in thermoplastic zein films modified with 5 wt % of MMT and using 25 wt % poly(ethylene glycol) as plasticizer. This loading of Z-MMT_S2 seems to act simultaneously as reinforcing filler and plasticizer in the zein matrix, reducing its brittleness even in the absence of glycerol. Similar results were observed in the STH/Z-MMT_S2 film, showing a Young’s modulus of 0.5 GPa, around twice that of the pristine starch film (0.2 GPa). This value is slightly higher than those reported for thermoplastic starch matrices reinforced by cationic starch-modified montmorillonite [27], probably because the addition of plasticizers was avoided in the current work. In contrast to the zein-based films, the positive effect of zein-based fillers on starch cannot be attributed to the compatibility between both systems, but it might be due to their plasticizing effect.

The elongation-at-break values of the bionanocomposites Z/Z-MMT_S2 and STH/Z-MMT_S2 were 2.0% and 1.98%, respectively, lower than those measured for neat zein and starch films (3.1% and 10.7%, respectively). These higher values in the blank biopolymer films are due to the glycerol plasticizer, as commented above, which increases flexibility and stretchability of the biopolymer matrix. In the bionanocomposite films, a reduced mobility of the zein and starch chains after incorporation of the biofiller can take place, as observed in other clay-reinforced polymer nanocomposites [39].

Although this preliminary study would require optimization for the preparation of bionanocomposites with better properties, these results point out to the promising use of zein-based biohybrids as an ecological alternative to conventional alkylammonium-modified clays usually employed as reinforcing agent.

## Conclusion

A new insight about the formation of biohybrids from the intercalation of zein protein in Na-montmorillonite clay was afforded comparing two methodologies of synthesis: the direct adsorption of zein from 80% (v/v) ethanol/water solution and the adsorption of the zein phases previously segregated in absolute ethanol to which is added a water suspension of the clay (80% (v/v) ethanol/water in the final system). Only the latter was more effective in achieving the incorporation of zein molecules into the intracrystalline space of Na-montmorillonite. A systematic study of the individual steps of intercalation of zein into the layered clay evidences that once zein phases are separated in ethanol, the ethanol-soluble components of zein first intercalate into Na-montmorillonite, being followed by a subsequent cooperative process in which the protein present in the biohybrid favors further adsorption of other zein components.

The obtained biohybrids were evaluated as bio-organoclays for the incorporation in zein and starch biopolymer films, which exhibited good compatibility, homogeneity and mechanical properties, and made it possible to avoid the addition of compatibilizers or plasticizers. These results suggest that these new biohybrid materials could be associated with other polymers of different nature, being a promising ecological alternative to common organoclays based on alkylammonium cations. Other fields of applying the biohybrids could be the use as biointerfaces of various biological species.

## Experimental

### Starting materials and reagents

A natural Wyoming sodium montmorillonite (MMT), commercialized as Cloisite^®^Na^+^, was purchased from Southern Clay Products (USA). Zein (Z) and starch from corn were purchased from Sigma-Aldrich, absolute ethanol from Panreac, and ninhydrin spray reagent 0.1% for chromatography from Merck. Deionized water (resistivity of 18.2 MΩ·cm) was obtained with a Maxima Ultrapure Water from Elga.

### Synthesis of zein–montmorillonite biohybrids

For the preparation of zein–montmorillonite biohybrids (Z-MMT) two synthetic routes were explored.

#### Synthesis 1 (Z-MMT_S1 materials)

A MMT suspension (6 g·L^−1^) was prepared in aqueous 80% (v/v) ethanol solution by vigorous stirring in a shear mixer (G2 model, Lomi) in order to properly disperse the clay. Solutions of zein (80% (v/v) ethanol/water) with different content in protein (30–1500 mg) were prepared in 50 mL, in order to achieve different weight proportions of zein with respect to montmorillonite in the biohybrid materials. Each zein solution was added to 50 mL of the MMT dispersion and the resulting mixture was stirred for 48 h at room temperature. Then, the solid product was isolated by centrifugation and dried overnight at 40 °C.

#### Synthesis 2 (Z-MMT_S2 materials)

In this synthesis, 300 mg of MMT were firstly swollen in 20 mL of water. Different amounts of zein (30–1500 mg) were added to 80 mL of ethanol. Zein was not completely dissolved in pure ethanol, but a separation process of different components of the protein took place, yielding an extracted phase (soluble in alcohol, denoted as EXT) and a solid or precipitate phase (insoluble in alcohol, denoted as PCT), resulting from the agglomeration of the insoluble components (Figure 1A). The aqueous clay suspension was then added to this system with the two phases of zein in absolute ethanol. The solid phase of the protein began to solubilize as the liquid phase reached a 80:20 ethanol/water ratio, forming at this point a homogeneous Z-MMT suspension. The system was kept under magnetic stirring for 48 h at room temperature, and then the solid was separated by centrifugation and dried overnight at 40 °C.

### Use of zein–montmorillonite as biofiller in bionanocomposites preparation

The biohybrid Z-MMT_S2_46 (i.e., containing 46 g of zein per 100 g clay) was used in the preparation of zein and starch films with different biofiller content (0, 1.25 and 3.50% with respect to the biopolymer mass). In the case of zein films loaded with zein–montmorillonite (Z/Z-MMT_S2), 2.5 g of zein were solubilized in 45 mL of aqueous ethanol solution (80% (v/v)), under vigorous magnetic stirring and kept at 80 °C. Then, 5 mL of zein–montmorillonite dispersion in water were added to the zein solution at 80 °C forming a single batch that was kept under stirring for approximately 30 min to reach room temperature. After total homogenization, the resulting dispersion was placed in a methacrylate mould, and dried at room temperature. The starch films based on zein–montmorillonite biohybrids (STH/Z-MMT_S2) were prepared similarly to the zein films discussed above, but starch was dissolved in pure water at 80 °C.

For comparison, zein and starch films containing MMT (Z/MMT and STH/MMT, respectively) were also prepared under the same conditions than those used for the preparation of the bionanocomposite films filled with biohybrids. Blank films of zein (Z) and starch (STH) were prepared by dissolving 2.5 g of zein or starch in 50 mL of ethanol solution at 80% (v/v) or pure water, respectively. In both systems it was necessary to add 0.5 g of glycerol as plasticizer, keeping the mixtures under magnetic stirring at 80 °C until complete homogenization of the components.

### Characterization

Fourier transform infrared (FTIR) spectra of samples in film form or diluted in KBr as pellets were recorded from 4000 to 250 cm^−1^ (2 cm^−1^ resolution) with a FTIR spectrophotometer BRUKER IFS 66v/S. CHNS elemental chemical microanalysis of samples was determined in a Perkin-Elmer 2400 analyzer. Solid-state CP-MAS ^13^C NMR spectra of samples spun at 10 kHz were obtained in a Bruker Avance 400 spectrometer, using a contact time of 2 ms and a period between successive accumulations of 5 s. The number of scans was 800 and chemical shift values were referenced to tetramethylsilane. The qualitative analysis of protein fractions (λ = 250–600 nm) and the UV–vis transmittance (λ = 200–800 nm) of bionanocomposite films (rectangular shape, 2 cm × 4 cm) were determined using a Shimadzu UV-1201 spectrophotometer. Surface morphology was observed with a FE-SEM equipment FEI-NOVA NanoSEM 230, which allowed semi-quantitative analysis of elements. The equipment allows for the direct observation of samples adhered on a carbon tape without requirement of any conductive coating on the surface. For the TEM images (Philips Tecnai 20, operating at 200 kV), the biohybrids were previously embedded in epoxy resin and then cut in very thin sections using an ultramicrotome (LEICA EM UC6) equipped with a diamond blade.

### SDS-PAGE

SDS-PAGE was performed according to Cabra and co-workers [29], where aliquots of 7.5 µL containing approximately 30 µg of zein solubilized in 80% (v/v) ethanol/water or those zein fractions obtained from pure ethanol were re-suspended in equal volumes of deionized water and buffer (0.125 M Tris-Cl, 4% SDS, 20% glycerol, 10% 2-mercaptoethanol (BME), and bromphenol blue 0.01%, pH 6.4). The EXT phase was directly used after separation in absolute ethanol, while the PCT phase was firstly solubilized in 80% (v/v) ethanol/water. The polyacrylamide gels at 20% were silver-stained for band visualization.

### Mechanical properties

The mechanical properties, Young’s modulus (*E*) and elongation at break, of the bionanocomposite film samples were evaluated (in three replicates) with a Model 3345 Instron Universal Testing Machine (Instron Engineering Corporation Canton, MA, USA) according to the ASTM standard method D 882-88. Rectangular samples (ca. 60 mm × 15 mm) were mounted between the grips with an initial separation of 50 mm, and the cross-head speed was set at 2 mm·min^−1^.

## Supporting Information

File 1 Additional experimental data.
