# Supplementary material for: Effective intercalation of zein into Na-montmorillonite: role of the protein components and use of the developed biointerfaces
Source: Beilstein J Nanotechnol. 2016 Nov 18;7:1772–82. doi: 10.3762/bjnano.7.170 (PMC5238646; doi:10.3762/bjnano.7.170)
Supplement: File 1 — Additional experimental data. [file Beilstein_J_Nanotechnol-07-1772-s001.pdf]

Supporting Information

for

Effective intercalation of zein into Na-montmorillonite: role of the protein components and use of the developed biointerfaces

Ana C. S. Alcântara[1,2], Margarita Darder*[1], Pilar Aranda*[1] and Eduardo Ruiz-Hitzky[1]

Address: [1]Instituto de Ciencia de Materiales de Madrid, CSIC, Cantoblanco, 28049-Madrid, Spain and [2]present address: Universidade Federal do Maranhão, Departamento de Química – PPGQuim, LIM-Bionanos, 65080-805, São Luís, MA, Brazil

Email: Margarita Darder - darder@icmm.csic.es; Pilar Aranda - aranda@icmm.csic.es

* Corresponding author

Additional experimental data

Zein characterization

The morphology of zein before and after dissolution in 80% (v/v) ethanol/water, as well as that of the phases separated from 18.7 mg/mL of zein dispersed in absolute ethanol and then dried in air was investigated by FE-SEM and several representative images are displayed in Figure S1. The commercial zein powder used in this work shows a compact and smooth texture formed by thick sheets (Figure S1a). However, after its solubilization in aqueous ethanol and subsequent air drying, the morphology of the protein varies giving rise to globular aggregates (Figure S1b), which may occur from aggregates of small size of around 176 nm up to approx. 4.0 μm. This morphology may result from the rearrangement of dissolved zein molecules during the drying process. The fast evaporation of ethanol may lead to a solution with an increasing content in water that contributes to enhance the zein–zein interactions, minimizing the number of hydrophobic chains exposed to water and, thus, reducing their undesirable interaction with the water molecules after ethanol evaporation (Wang, Y.; Padua, G. W. *Langmuir* **2010,** *26,* 12897). This texture differs from that of the EXT phase recovered after treatment of zein with ethanol which shows certain molecular aggregates, slightly distorted in this case, presenting also some plate-like shape agglomerates (indicated by white arrows in Figure S1c). In contrast to the typical zein morphology, the PCT fraction does not show agglomerates (Figure S1d), forming a continuous and homogeneous film of compact texture, very similar to the commercial zein morphology (Figure S1a). These different morphologies in zein can be associated with the relative amount of protein components in each one of the separated phases. This interpretation agrees with another study reporting the effect of zein concentration on the

texture adopted by the protein when it is dried from ethanol-water dispersion (Wang, Y.; Padua, G. W. *Langmuir* **2010,** *26,* 12897) Thus, diluted zein solutions showed the formation of free microspheres occasioned by hydrophobic associations, which is the case of the EXT phase. In contrast, zein recovered from solution at higher concentration in alcohol has a tendency to form narrow packed aggregates that subsequently fuse into a film during the drying process, which may be a similar situation to that of the PCT fraction in the current study.

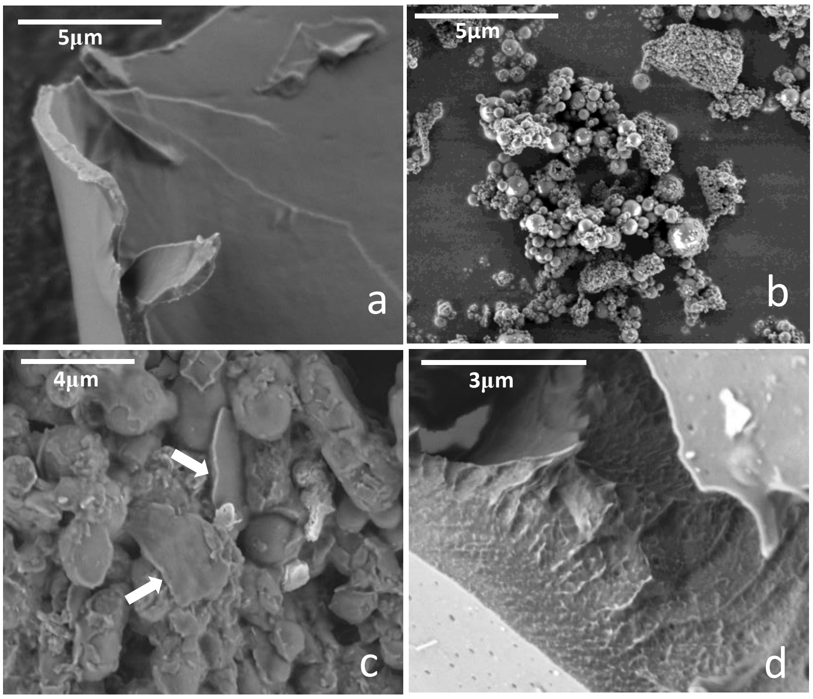

Figure S1: FE-SEM images of (a) commercial pristine zein powder and (b) zein recovered after drying a protein solution in 80% (v/v) ethanol/water; (c) EXT and (d) PCT.

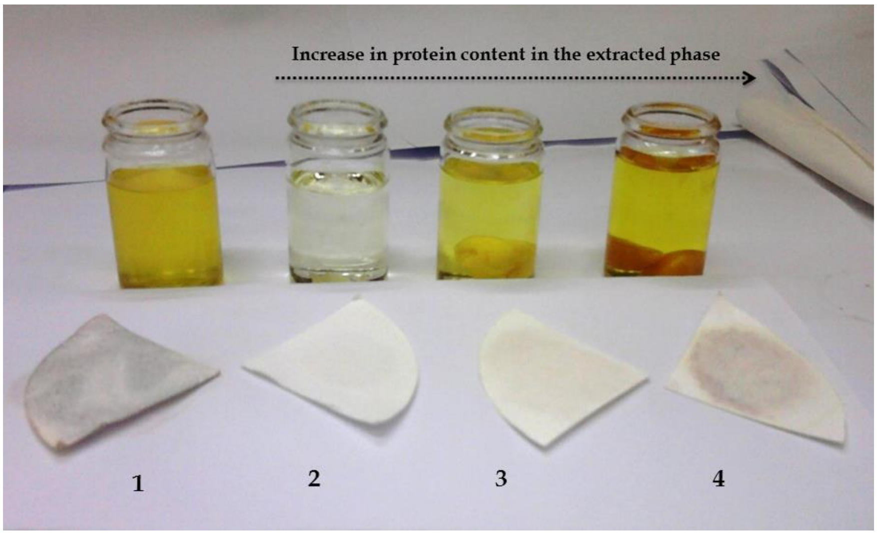

Figure S2: Colorimetric assay using ninhydrin spray solution as protein revealing agent in different zein preparations: (1) 0.3% (w/v) zein solution in 80% (v/v) ethanol/water as reference, and (2–4) zein in pure ethanol in 0.018, 0.15 and 0.3% (w/v) concentration.

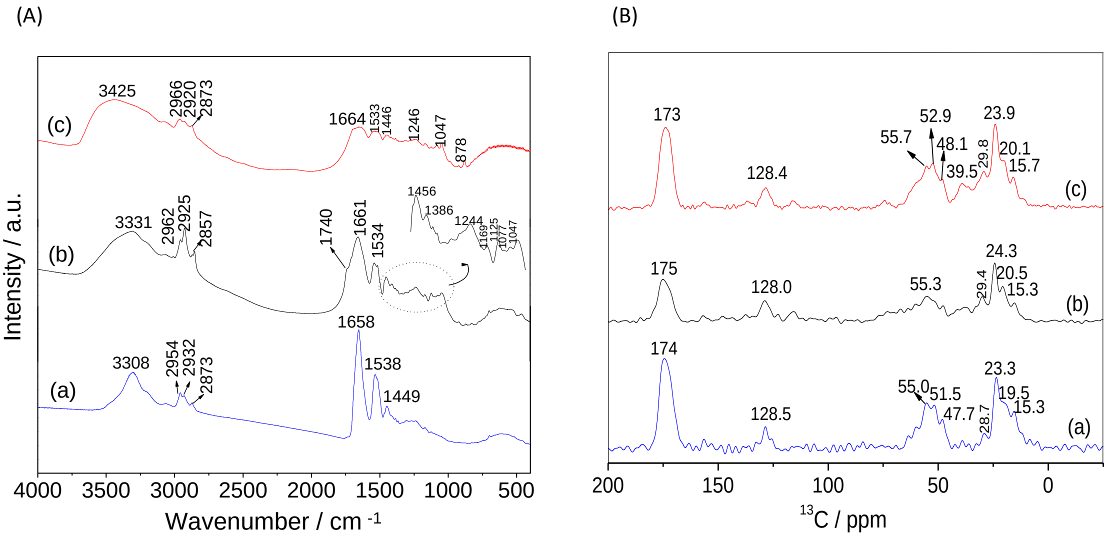

Figure S3: (A) FTIR spectra in the 4000–400 cm^{-1} region and (B) ^{13}C NMR spectra of (a) zein protein, and those of the (b) extracted phase and (c) precipitate fraction of zein after treatment in pure ethanol.

Zein–montmorillonite bio-hybrids

The affinity between the protein and MMT via the synthesis route 2 was also investigated by analyzing the "pseudo-isotherms" of this adsorption process at 23 °C represented in Figure S4. In contrast to the synthesis route 1 (Figure S4a), the shape of this "isotherm" indicates that zein present in the system was almost completely adsorbed at low initial concentration, which resembles to classical H-type isotherm (Giles, C. H.; MacEwan, T. H.; Nakhwa, S. N.; Smith, D. *Journal of the Chemistry Society* **1960,** *111,* 3973). This "pseudo isotherm" shows a plateau region at zein equilibrium concentration above 2.0 $g{\cdot}L^{-1}$, corresponding to approximately 40 g of zein adsorbed per 100 g of montmorillonite. At these values of adsorbed zein, a monolayer-type conformation of the zein molecules into MMT is expected.

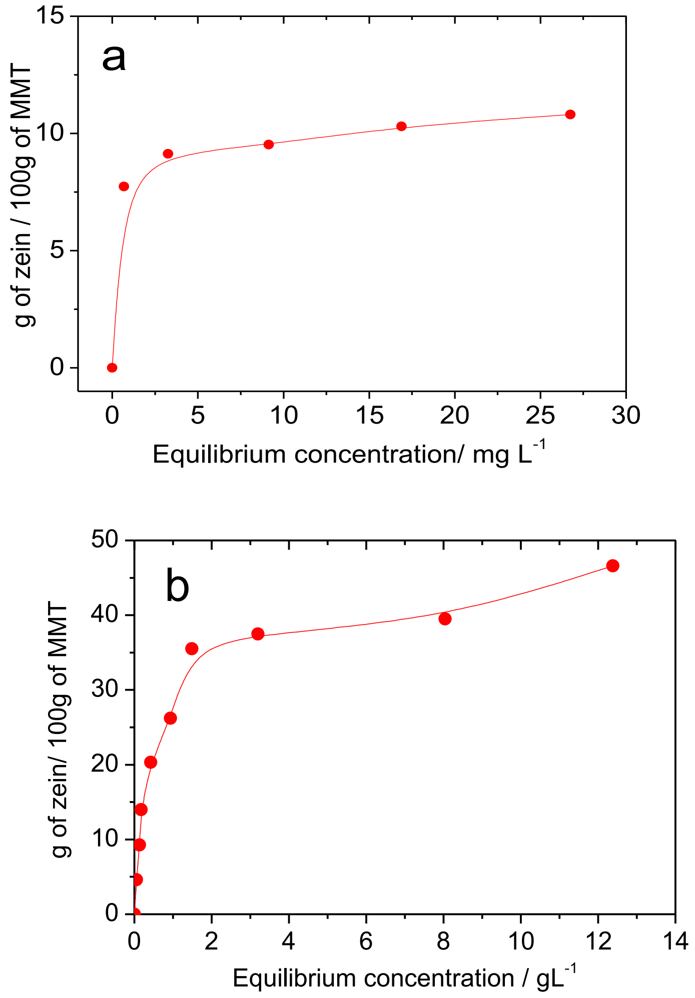

Figure S4: Adsorption profiles at 23 °C of zein (a) from 80% (v/v) ethanol/water solution (synthesis 1) and (b) from zein in pure ethanol at 23 °C after mixture with MMT water suspension to reach an 80% (v/v) ethanol/water concentration (synthesis 2). Adsorption amounts were deduced from CHNS chemical analyses.

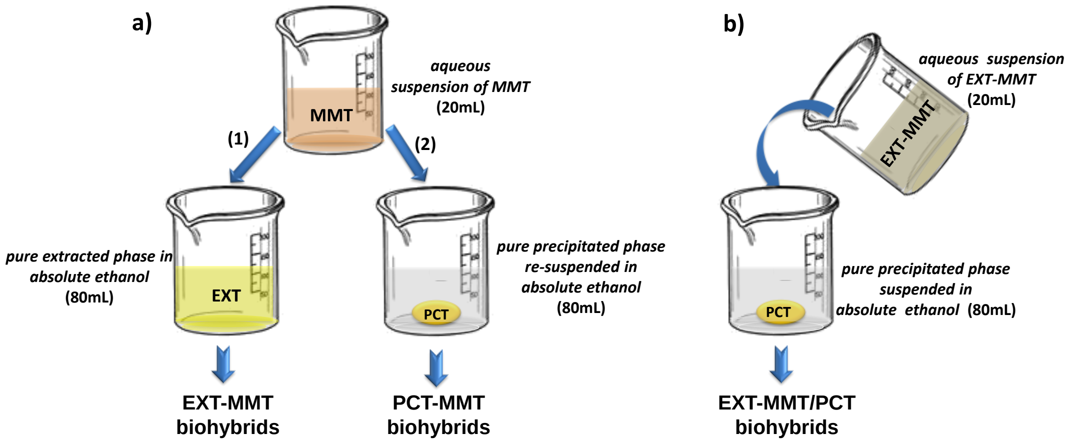

Figure S5: (a) Sequence of individual steps followed to produce zein intercalation into MMT from (1) the protein extracted phase in pure ethanol, and (2) the precipitate phase after re-suspended in absolute ethanol, and mixing with a water suspension of the clay; (b) Preparation of EXT-MMT/PCT biohybrids by mixing an aqueous dispersion of EXT-MMT biohybrids with PCT re-suspended in pure ethanol.

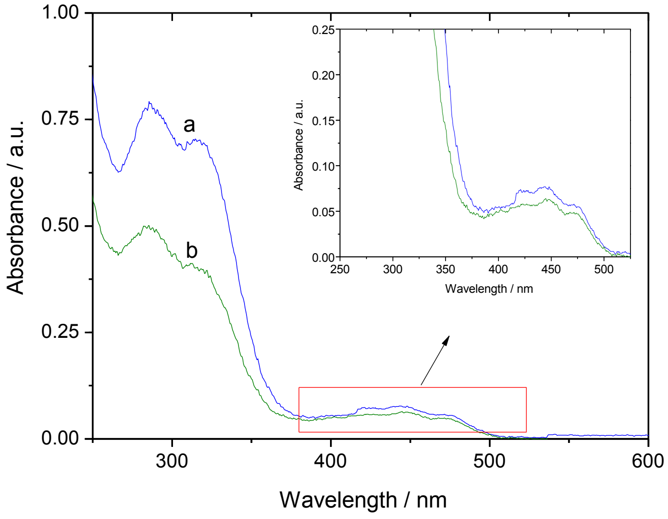

Figure S6: UV–vis (250–600 nm) of (a) extracted phase from zein in pure ethanol and (b) the supernatant of EXT-MMT biohybrid.

More insights on the compatibility of these bionanocomposite films were obtained from UV–vis transmittance measurements in the wavelength range between 200 and 800 nm (Figure S7). The transmittance values for zein films involving Z-MMT_S2 biohybrid were higher than those of zein films loaded with neat MMT, which showed very low transparency with transmittance values around 1.7% for the film loaded with 3.5% (w/w) of MMT (Figure S7a). In contrast, transmittance values of Z/Z-MMT_S2-1.25% ranged between 60% and 75%, pointing out to a better compatibility of the Z-MMT biohybrid with the zein matrix.

On the other hand, all the starch bionanocomposite films (Figure S7b) show high transmittance values in the visible region (above 70%), indicating a high degree of transparency, and the lowest values of light transmission are observed for the bionanocomposite film loaded with 3.5% (w/w) of Z-MMT_S2 biohybrid. In contrast to zein-based systems, the transparency of starch films loaded with neat MMT is quite similar to that of the blank film, indicating that the clay is better dispersed within the starch matrix, possibly due to the higher hydrophilic character of this polymer that facilitates its compatibility with the neat clay.

In comparison to the films prepared with pure biopolymers, all the bionanocomposites show good barrier properties in the UV region, with a remarkable reduction of transmission in the case of starch.

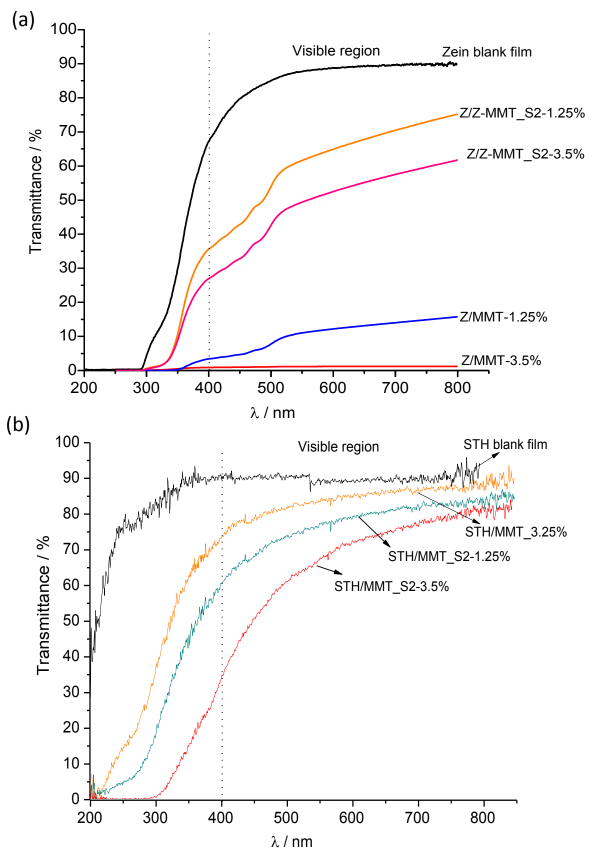

Figure S7: Transmittance measurements in the 200–800 nm region of (a) zein and (b) starch bionanocomposite films loaded with 1.25 and 3.5% (w/w) of MMT or Z-MMT_S2 biohybrid.
